# Development of Microsatellite Marker System to Determine the Genetic Diversity of Experimental Chicken, Duck, Goose, and Pigeon Populations

**DOI:** 10.1155/2021/8851888

**Published:** 2021-01-14

**Authors:** Xiulin Zhang, Yang He, Wei Zhang, Yining Wang, Xinmeng Liu, Aique Cui, Yidi Gong, Jing Lu, Xin Liu, Xueyun Huo, Jianyi Lv, Meng Guo, Xiaoyan Du, Lingxia Han, Hongyan Chen, Jilan Chen, Changlong Li, Zhenwen Chen

**Affiliations:** ^1^Capital Medical University, School of Basic Medical Sciences, Beijing 100069, China; ^2^Harbin Veterinary Research Institute of Chinese Academy of Agricultural Sciences, Harbin 150069, China; ^3^Institute of Animal Sciences, Chinese Academy of Agricultural Sciences, Beijing 100193, China

## Abstract

Poultries including chickens, ducks, geese, and pigeons are widely used in the biological and medical research in many aspects. The genetic quality of experimental poultries directly affects the results of the research. In this study, following electrophoresis analysis and short tandem repeat (STR) scanning, we screened out the microsatellite loci for determining the genetic characteristics of Chinese experimental chickens, ducks, geese, and pigeons. The panels of loci selected in our research provide a good choice for genetic monitoring of the population genetic diversity of Chinese native experimental chickens, ducks, geese, and ducks.

## 1. Introduction

Laboratory animals are important experimental materials for science research. They play key roles in the investigation of pathogenesis, diagnosis of diseases, pharmaceutical research, and other fields [1]. The genetic quality of laboratory animals directly affects the accuracy, repeatability, and scientificity of medical biological research results. Genetic monitoring is one of the effective methods to evaluate population's genetic diversity. Through genetic monitoring, whether genetic mutations and genetic pollution occurred can be analyzed.

Poultry, including chicken, duck, goose, and pigeon, has become commonly used laboratory animals [2]. They are easy to reproduce and hatch in vitro. Among them, chickens are the most widely used poultry in life science research [3, 4]. Ducks, geese, and pigeons also play important roles in the research of epidemiology, immunology, virology, and pharmacotoxicology [5–9]. There are many genetic analysis and quality control methods applied to chickens [10, 11]. However, at present, we find few reports about the genetic analysis systems and quality control methods of duck, goose, and pigeon populations, especially in the Chinese native groups.

Hence, in this study, we screened out the microsatellite loci with uniform distribution, stable amplification, and rich polymorphism in experimental chickens, ducks, geese, and pigeons with different genetic backgrounds [12]. We developed effective microsatellite marker systems to determine the genetic diversity of experimental chickens, ducks, geese, and pigeons, which will lay the foundation for the genetic quality control of them and promote the application of experimental poultry.

## 2. Materials and Methods

### 2.1. Animal Sample

Three outbred groups and three haplotype groups of experimental chicken were used in this research: outbred group BWEL-SPF chickens ((SCXK (black) 2017-005)), 40 samples, 37 weeks old, 6 males and 34 females, which has been closed for 20 generations; outbred group BM chicken (from BWEL chicken lineage (SCXK (black) 2017-005)), 40 samples, 14 weeks old, 6 males and 34 females; outbred group Beijing oil chickens, 46 samples. MHC haplotype chickens were bred from the 13th generation of BWEL chicken, the haplotype was continuously selected based on the MHC core genes, and the half-sibling or sibling mating method was used to breed to the 8th generation [13]. We selected 5 G1 haplotype chickens, 53 weeks old, 1 male and 4 females; 5 G2 haplotype chickens, 93 weeks, 1 male and 4 females; and 5 G7 haplotype chickens, 82 weeks, 1 male and 4 females. The Beijing oil chickens came from the Institute of Animal Science (IAS), Chinese Academy of Agricultural Sciences (CAAS). Other samples were from Harbin Veterinary Research Institute (HVRI), CAAS. All the samples were blood.

Two outbred groups and four haplotype groups of experimental duck (bred from Jinding (JD) duck lineage (SCXK (black) 2017-006)) were selected: outbred group 1, 40 samples, 37 weeks old, 6 males and 34 females; outbred group JD duck, 40 samples, 37 weeks old, 6 males and 34 females; 10 A haplotype ducks, 53 weeks old, 1 male and 4 females; 10 B haplotype ducks, 53 weeks old, 1 male and 4 females; 10 C haplotype ducks, 53 weeks old, 1 male and 4 females; 10 D haplotype ducks, 53 weeks old, 1 male and 4 females. All the samples are duck muscle tissue and were from HVRI, CAAS.

We collected two outbred groups of experimental geese: outbred group Guangdong Wuzong goose, 44 samples, 37 weeks old, 6 males and 34 females; outbred group Yangzhou goose, 44 samples, 37 weeks old, 6 males and 34 females. All the samples are goose liver tissue. Guangdong Wuzong geese were from Southern Medical University, and Yangzhou geese were from Yangzhou University.

Forty pigeons were randomly selected from two populations of white king pigeons and silver king pigeons, half male and half female, with no age limit. All the animals were from Liujinlong pigeon farms in Beijing. Their heart tissues were collected.

All breeding is carried out in accordance with Chinese agricultural standards NY/T 1901. What is more, all experiments followed the 3R principle.

### 2.2. Microsatellite Locus Selection

By searching PubMed and using SSR Hunter software to analyze animal gene information, we obtained microsatellite loci for further screening.

### 2.3. DNA Extraction

Phenol-chloroform extraction method was used to extract DNA from muscle, liver, and heart tissue. TIANamp Blood DNA Kits (Tiangen, Beijing, China) were used to extract DNA from chicken blood samples. All DNA concentrations were diluted to 50 ng/*μ*L, stored in -20°C.

### 2.4. PCR Procedure and Agarose Gel Electrophoresis

The PCR was performed in a 20 *μ*L reaction volume containing 10 *μ*L Dream Taq Green PCR Master Mix (Thermo Fisher Scientific, Massachusetts, MA), 2 *μ*L pure water (ddH_2_O), 10 pmol each primer, and 50 ng of the extracted DNA template. The PCR protocol was as follows: 94°C for 5 min, followed by 35 cycles of 94°C for 30 s, suitable temperature for 30 s, 72°C for 30 s, and a final extension at 72°C for 5 min. Amplified products were stored at -20°C for further analysis.

Amplified products were electrophoresed on a 2% agarose gel at 130 V, 90 min.

### 2.5. STR Scanning

We performed STR scanning on PCR amplification products of candidate loci. The forward primers of candidate microsatellite loci were fluorescent labelled with FAM, HEX, and TAMRA. The sample genome was amplified with fluorescent primers, and the amplified products were scanned by STR through 3730xl DNA Analyzer (Applied Biosystems, Thermo Fisher Scientific, Massachusetts, USA). All the STR scanning was performed by Beijing Tianyi Huiyuan Biotechnology Co., Ltd.

### 2.6. Data Analysis

GeneMarker V2.2.0 software was used to analyze the length of amplified fragments from different populations at each microsatellite locus. Popgene 3.2 software was used to analyze the observed number of alleles, effective number of alleles, Shannon's information index, and effective heterozygosity of microsatellite loci. The polymorphic information content of multiple sites was calculated using PIC calculation software (PIC_CALC.0.6).

## 3. Results

### 3.1. Microsatellite Locus Selection

#### 3.1.1. Preliminary Screening of Microsatellite Loci by PCR

Firstly, we obtained the microsatellite locus information of experimental chickens, ducks, geese, and pigeons by searching previous reports on PubMed and using the SSR Hunter software to analyze the genetic information of different populations [14, 15]. We collected 72, 59, 57, and 61 microsatellite loci of experimental chicken, duck, goose, and pigeon, respectively.

**Figure 1:**
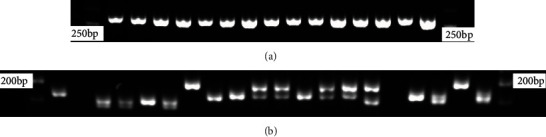
Results of agarose gel electrophoresis of microsatellite DNA locus GGNCAMZO in experimental chickens and locus AY264 in experimental ducks. (a) GGNCAMZO in haplotype chicken line G1. (b) AY264 in the outbred group of experimental ducks.

In order to clarify the amplification conditions of the microsatellite loci and exclude the loci with poor specificity, we performed temperature gradient PCR and agarose gel electrophoresis of microsatellite loci. Then, we performed PCR amplification on the most suitable conditions and subjected the PCR products to agarose gel electrophoresis to screen out loci with suitable length, good polymorphism in outbred groups, good monomorphism in haplotypes, and high specificity. Taking the chicken GGNCAMZO locus and duck AY264 locus as example, the results are shown in Figure 1. GGNCAMZO locus is monomorphic in the haplotype chicken population, and AY264 locus is polymorphic in the outbred duck group.

In summary, we selected 37 and 32 microsatellite loci with good polymorphism in the outbred groups and haplotypes of chicken, respectively [12, 16, 17]. In addition, 15 and 23 loci were screened in the outbred groups and haplotypes of duck, respectively [14, 18, 19]. In the outbred groups of goose and pigeon, 14 and 20 microsatellite loci were chosen [18, 20–23]. Loci in these panels would be candidate for the final microsatellite marker evaluation systems.

#### 3.1.2. STR Scanning Analysis

**Figure 2:**
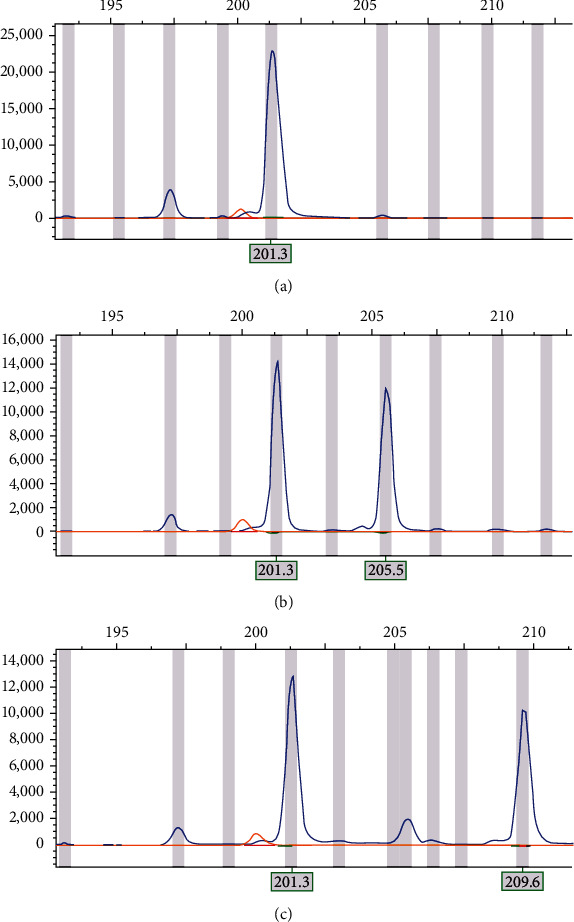
Results of UU-Cli*μ*T47 scan of the experimental pigeons. (a) The STR graph corresponding to the sample of haplotype under primer UU-Cli*μ*T47 shows homozygote with a wave peak of 201 bp. (b) The STR diagram corresponding to the sample of outbred groups under primer UU-Cli*μ*T47 shows heterozygote with two wave peaks of 201 bp and 205 bp, respectively. (c) The STR diagram corresponding to the sample of outbred groups under primer UU-Cli*μ*T47 shows heterozygote with two wave peaks of 201 bp and 209 bp, respectively.

In order to further complete the microsatellite marker system, we performed STR scanning on the candidate microsatellite DNA loci matched microsatellite criteria and analyzed the length of the amplified product at the peak with GeneMarker software (V1.75). Taking the UU-Cli*μ*T47 locus as an example, it showed polymorphism in the outbred group of pigeon (Figure 2).

We finally determined that in experimental chickens, 28 loci were selected for genetic monitoring in the outbred groups and 14 loci for haplotypes. All microsatellite DNA loci are shown in Table 1. There are 13 common loci.

In experimental duck populations, we chose 25 loci and 15 loci for genetic monitoring in the outbred duck groups and haplotype groups. There are 12 common loci. Microsatellite loci are shown in Table 2.

14 microsatellite loci with good polymorphism were considered as microsatellite markers in the outbred group of goose.  Table 3 demonstrates the number of alleles, optimal amplification conditions, and fragment length of 14 alleles for the outbred experiment geese.

In the outbred group of pigeon, we finally screened out 16 microsatellite loci with good polymorphism, several alleles, and typical stutter peaks. All microsatellite locus information is shown in Table 4.

#### 3.1.3. Analysis of Population Microsatellite Loci

We inputted the results of STR scanning into Popgene 3.2 to analyze experimental chicken in the outbred groups and the haplotypes at 29 loci. In the outbred groups, 28 microsatellite loci show a high degree of polymorphism, and the average number of observed alleles is 4.571. The average number of effective alleles is 3.270, and the average Shannon's information index is 1.198 (Table 5). Furthermore, the average effective heterozygosity is 0.492. The average polymorphism information content (PIC) is 0.610. All these data indicate a good genetic diversity of screening loci in the outbred groups and large heterozygosity difference among the laboratory experimental chicken populations.

In the other 3 haplotype populations, 14 microsatellite loci showed monomorphism in each population but showed different lengths in different haplotype populations. The average number of observed alleles is 1.571. The average number of effective alleles, the average Shannon's information index, and the average effective heterozygosity are 1.433, 0.316, and 0.207, respectively (Table 6). The specific data of each haplotype population is shown in Supplementary Tables 1–3.

In the outbred group of duck, 25 microsatellite loci show polymorphism. The average number of observed alleles is 7.520, and the average number of effective alleles in the population is 4.162. The average Shannon's information index is 1.574, and the average effective heterozygosity is 0.683. The average PIC is 0.698. These data showed that in the outbred groups, the genetic diversity of microsatellite DNA loci is better, and the genetic diversity of each locus is quite different. The specific results are shown in Table 7.

In 4 haplotype populations, 15 microsatellite loci show monomorphism in each population. The average number of observed alleles is 4.133, the average number of effective alleles is 2.863, and the average Shannon's information index is 1.153, indicating that the genetic diversity of the loci in these haplotype populations is poor; the average effective heterozygosity is 0.500, indicating that the heterozygosity difference is small and the genetic information of the selected loci is relatively single. See Table 8 for more detailed information, and the specific data in each haplotype population is shown in Supplementary Tables 4–7.

In the outbred colony of experimental goose, 14 loci were selected. The average number of observed alleles, the average number of effective alleles, the average Shannon's information index, the average effective heterozygosity, and the PIC are 4.714, 3.038, 1.195, 0.528, and 0.582, respectively. The microsatellite loci have large interindividual differences within the population, and the population has high gene stability (Table 9).

The selected microsatellite loci all show good polymorphism in the experimental outbred pigeon populations. A total of 16 loci were selected. The average number of observed alleles is 7.875. The average effective allele number is 4.554; the average Shannon's information index and the average effective heterozygosity are 1.559 and 0.649. The average PIC is 0.674 (Table 10).

#### 3.1.4. Population Genetic Structure Analysis

Among the three outbred chicken groups, the mean number of observed alleles, the mean number of effective alleles, the mean Shannon's information index, and the mean effective heterozygosity are shown in Table 11. All these data are the highest in the Beijing oil chicken, indicating the best gene diversity.

In the haplotype chicken populations, the highest mean observed number of alleles is observed in G7groups. Haplotype G7 has the highest mean effective allele number and the highest mean Shannon's information index. The mean effective heterozygosity of haplotype G7 is 0.364. The genetic heterozygosity of the 3 populations is very low, and the consistency is good (Table 12).

In the two outbred groups of duck, the mean number of observed alleles, the mean effective number of alleles, the mean Shannon's index, and the mean effective heterozygosity of outbred group 1 are higher than those of outbred group JD, indicating that outbred group 1 had better diversity. The results are shown in Table 13. Among the 4 haplotype populations, the highest mean number of alleles is observed in haplotype A. Haplotype A has the highest mean Shannon's information index. The highest mean effective heterozygosity in the duck groups is 0.489 in haplotype A (Table 14). The genetic heterozygosity of 4 populations is in good agreement.

In the two outbred groups of goose, the mean number of observed alleles, the mean effective number of alleles, and the mean Shannon's index of Guangdong Wuzong goose are higher than those of Yangzhou goose, indicating that Guangdong Wuzong goose has a better diversity (Table 15).

The analysis of the two main experimental pigeon populations used for scientific research shows that the mean effective heterozygosity of two populations is 0.647 and 0.651, respectively. The mean number of observed alleles, the mean effective number of alleles, and the mean Shannon's index are higher in white king pigeons than in silver king pigeons. The comparison of the data is shown in Table 16.

## 4. Discussion

Poultries are widely used and are indispensable supporting conditions for the life sciences and biomedicine industries. Specific pathogen-free (SPF) chicken embryos are used in the manufacture and quality control of biological product [4]; ducks play an important role in the research of avian influenza, fatty liver, duck hepatitis A, and duck hepatitis B [5–7]; goose blood contains a higher concentration of immunoglobulin, which is often used in pharmacology and toxicology research [8]; pigeons belong to the class of birds and are considered as important animal model in avian influenza research [9]. With the increasing demand for experiment poultry, people are paying more attention to the genetic structure analysis and genetic quality control. However, the current methods of genetic structure analysis and genetic quality control for experimental poultry animals are insufficient.

Coat colour gene testing method, biochemical marker gene testing method, immune marker gene testing method, and DNA molecular marker method are popular methods for genetic monitoring. Microsatellite DNA, mitochondrial DNA (mtDNA), restriction fragment length polymorphism (PCR-RFLP), single-stranded conformation polymorphism (PCR-SSCP), and specific gene polymorphisms are commonly used DNA molecular marker methods [24–27]. Among them, microsatellite DNA has become valuable tools for evaluating population genetic diversity due to their unique virtue.

Microsatellite DNA is characterized by short tandem repeats (STRs) of 1 to 6 nucleotides in eukaryotic genome with a random manner [28]. It has rich polymorphism and large genetic information. Microsatellite can be used to distinguish heterozygous from homozygous because of their codominant inheritance feature [29]. In previous studies, microsatellites have been used as biomarkers for monitoring rodent genetic traits [30, 31]. With the deep understanding of microsatellites, it plays a more important role in genetic monitoring for being simple, clear, and stable in operation. In this research, we screened out microsatellite loci with suitable length and high specificity as candidate loci by gel electrophoresis firstly. Then, we performed STR scanning on these candidate loci. Microsatellite loci with good polymorphism, abundant alleles in the outbred groups, and good monomorphism in the haplotype populations were selected to form the microsatellite marker system. We analyzed the average effective allele number, average Shannon's index, average effective heterozygosity, and other analytical indices to estimate genetic variation in different groups.

The mean effective number of alleles is an indicator of genetic variation and mutation drift balance. In our study, Beijing oil chicken has the highest mean effective allele number of three outbred chicken populations; outbred duck group 1 has higher mean effective allele number than outbred duck group JD. The outbred goose group Guangdong Wuzong and outbred pigeon group white king have the highest mean number of effective alleles in outbred goose populations and outbred pigeon populations, respectively. The higher mean effective number of alleles indicates that the population can maintain the original gene and avoid new variations under the pressures from genetic drift and artificial selection. The results show that Beijing oil chicken, outbred duck group 1, Guangdong Wuzong goose, and white king pigeon are the most stable strains in the outbred group of experiment chicken, duck, goose, and pigeon groups in this research, respectively.

The mean effective heterozygosity of a population is an important indicator of population genetic diversity and can reflect the richness of the detected genes. It is generally believed that when the mean effective heterozygosity of the population is less than 0.5, it indicates that the individual differences in the population are small and the genetic heterozygosity is low, which does not conform to the genetic characteristics of an outbred group animal. When the mean effective heterozygosity of the population is higher than 0.7, its genetic diversity is high [32].

Hence, we found that the mean effective heterozygosity of BWEL, BM, and Beijing oil chicken groups is all greater than 0.5, which conforms to the characteristics of the outbred group. The mean effective heterozygosity of BWEL and BM chicken groups is nearly 0.5. The average effective heterozygosity of G1, G2, and G7 groups is all less than 0.5. It is also consistent with the background that BWEL, BM, and Beijing oil chickens are outbred colonies; Beijing oil chicken has abundant genetic diversity and high selection potential for it has the highest mean effective heterozygosity among the outbred chicken groups in this study. This may be due to the large population. Duck group 1 and JD duck all have a mean effective heterozygosity greater than 0.680 which indicates a high genetic diversity. The mean effective heterozygosity of Guangdong Wuzong goose group, silver king pigeon group, and white king pigeon group is all greater than 0.5 which reflects abundant genetic diversity. The mean effective heterozygosity of three haplotype chicken groups and four haplotype duck groups is 0.207 and 0.500, respectively. The result indicates a good consistency in haplotype chickens and ducks. This may be the result of long-term full-sib and half-sib reproduction. Chickens and ducks are more widely used in biological research, and the breeding standards are stricter, while geese and pigeons are more useful in agriculture. Haplotype chickens have lower mean effective heterozygosity than haplotype duck populations, which is consistent with a longer history of breeding in experimental chickens.

When measuring the degree of gene variation, PIC is often used as a variation index. It is generally believed that when PIC is between 0.25 and 0.5, it is moderately polymorphic, and <0.25 shows a low level of polymorphism, when PIC is greater than 0.5, it means a high level of polymorphism [33]. In our microsatellite marker system, most of the microsatellite sites have a PIC greater than 0.5 that show high polymorphism. All these data prove that our microsatellite marker system provides rich genetic information, which can be used as effective genetic markers. In our study, highly polymorphic microsatellite marker systems showed powerful markers for quantifying genetic variations within and between poultry populations. We will collect more samples to make a more accurate description of genetic structure of the Chinese experimental chickens, ducks, geese, and pigeons in the future [34].

## 5. Conclusions

In conclusion, we identified appropriate microsatellite marker systems for native experimental chickens, ducks, geese, and pigeons in China. The combination of loci selected in our research provides a good choice for genetic monitoring of the quality and the population genetic diversity of poultry stocks.

## Figures and Tables

**Table 1:** Number of alleles, optimal amplification conditions, and fragment length of 29 alleles for the laboratory chickens.

Loci	Primer sequence (5′-3′)	Temperature(°C)	Allele range	Applicable groups
MCW0029	GTGGACACCCATTTGTACCCTATG	63.8	139-188	Outbred group
	CATGCAATTCAGGACCGTGCA			
ADL0293	GTAATCTAGAAACCCCATCT	53.9	106-120	Outbred group
	ACATACCGCAGTCTTTGTTC			
ADL0317	AGTTGGTTTCAGCCATCCAT	58.5	177-219	Outbred group
	CCCAGAGCACACTGTCACTG			
GCT0016	TCCAAGGTTCTCCAGTTC	52.2	111-148	Outbred group
	GGCATAAGGATAGCAACAG			
ADL0304	GGGGAGGAACTCTGGAAATG	53.9	138-161	Outbred group
	CCTCATGCTTCGTGCTTTTT			
LEI0074	GACCTGGTCCTGACATGGGTG	58.5	221-243	Outbred group
	GTTTGCTGATTAGCCATCGCG			
ADL328	CACCCATAGCTGTGACTTTG	53.9	107-120	Outbred group
	AAAACCGGAATGTGTAACTG			
GGANTECl	GCGGGGCCGTTATCAGAGCA	65.0	139-194	Outbred group
	AGTGCAGGGCGCTCCTGGT			
LEI094	CAGGATGGCTGTTATGCTTCCA	56.0	176-211	Outbred group
	CACAGTGCAGAGTGGTGCGA			
MCW0330	TGGACCTCATCAGTCTGACAG	58.5	217-287	Outbred group
	AATGTTCTCATAGAGTTCCTGC			
LEI0141	CGCATTTGATGCATAACACATG	52.2	221-245	Outbred group
	AAGGCAAACTCAGCTGGAACG			
MCW0087	ATTTCTGCAGCCAACTTGGAG	58.5	268-289	Outbred group
	CTCAGGCAGTTCTCAAGAACA			
MCW0347	GCTTCCAGATGAGCTCCATGG	52.0	121-149	Outbred group
	CACAGCGCTGCAGCAACTG			
ADL176	TTGTGGATTCTGGTGGTAGC	58.5	183-200	Outbred group
	TTCTCCCGTAACACTCGTCA			
ADL0201	GCTGAGGATTCAGATAAGAC	58.5	111-151	Outbred group
	AATGGCYGACGTTTCACAGC			
GGNCAMZO	GTCACTAGGTTAGCAGCATG	56.0	234	Outbred group
	GCTGGATACAGACCTCGATT			Haplotype
GGAVIR	AGAGATGGTGCACGCAACCT	60.7	86-89	Outbred group
	CGAGCACTTTCTGGCAGAGA			Haplotype
MCW0063	GGCTCCAAAAGCTTGTTCTTAGCT	53.9	116-146	Outbred group
	GAAAACCAGTAAAGCTTCTTAC			Haplotype
ADL185	CATGGCAGCTGACTCCAGAT	58.5	116-142	Outbred group
	AGCGTTACCTGTTCGTTTGC			Haplotype
GGMYC	CGAGGCGCTCTGCGAGTTTA	62.4	139-151	Outbred group
	TGGGGACCTCTGGCTCTGAC			Haplotype
LEI0094	GATCTCACCAGTATGAGCTGC	53.9	250-283	Outbred group
	TCTCACACTGTAACACAGTGC			Haplotype
GGVITC	AGCCATCATTCAGGGCATCT	58.5	86	Outbred group
	GATGTCCTGAGTGATGCTCA			Haplotype
ADL0292	CCAAATCAGGCAAAACTTCT	58.5	110-136	Outbred group
	AAATGGCCTAAGGATGAGGA			Haplotype
GGVITIIG	GGCAGGTTTCTAATGCCTGA	56.0	186-189	Outbred group
	CCCATCGTTTCAACTGTATG			Haplotype
ADL166	TGCCAGCCCGTAATCATAGG	58.5	131-154	Outbred group
	AAGCACCACGACCCAATCTA			Haplotype
MCW0014	AAAATATTGGCTCTAGGAACTGTC	58.5	172-195	Outbred group
	ACCGGAAATGAAGGTAAGACTAGC			Haplotype
GGCYMA	AGCGAGGCGCTCTGCGAGTT	64.6	140-153	Outbred group
	GGGCACCTCTGGCTCTGACC			Haplotype
MCW0402	ACTGTGCCTAGGACTAGCTG	56.0	141-229	Outbred group
	CCTAAGTCTGGGCTCTTCTG			Haplotype
STMSGGHU2-1A	CTTAATATGTGTGAGGTGGC	53.9	235-238	Haplotype
	GTTCTCACAATTGCATTAGC			

**Table 2:** Number of alleles, optimal amplification conditions, and fragment length of 28 alleles for the laboratory ducks.

Loci	Primer sequence(5′-3′)	Temperature (°C)	Allele range	Applicable groups
CAUD007	ACTTCTCTTGTAGGCATGTCA	60.8	100-190	Outbred group
	CACCTGTTGCTCCTGCTGT			
CAUD004	TCCACTTGGTAGACCTTGAG	60.8	234-385	Outbred group
	TGGGATTCAGTGAGAAGCCT			
CAUD023	CACATTAACTACATTTCGGTCT	51.4	163-234	Outbred group
	CAGCCAAAGAGTTCAACAGG			
CAUD027	AGAAGGCAGGCAAATCAGAG	66.0	70-180	Outbred group
	TCCACTCATAAAAACACCCACA			
CAUD001	ACAGCTTCAGCAGACTTAGA	55.5	150-247	Outbred group
	GCAGAAAGTGTATTAAGGAAG			
CAUD031	AGCATCTGGACTTTTTCTGGA	51.4	107-187	Outbred group
	CACCCCAGGCTCTGAGATAA			
CAUD032	GAAACCAACTGAAAACGGGC	58.1	96-206	Outbred group
	CCTCCTGCGTCCCAATAAG			
AY314	CTCATTCCAATTCCTCTGTA	50.3	112-329	Outbred group
	CAGCATTATTATTTCAGAAGG			
CMO211	GGATGTTGCCCCACATATTT	55.0	112-205	Outbred group
	TTGCCTTGTTTATGAGCCATT			
APH09	GGATGTTGCCCCACATATTT	58.0	134-190	Outbred group
	TTGCCTTGTTTATGAGCCATTA			
APH11	GGACCTCAGGAAAATCAGTGTA	58.5	183-185	Outbred group
	GCAGGCAGAGCAGGAAATA			
APL2	GATTCAACCTTAGCTATCAGTCTCC	58.5	115-125	Outbred group
	CGCTCTTGGCAAATGTCC			
CAUD011	TGCTATCCACCCAATAAGTG	50.3	145-223	Outbred group
	CAAAGTTAGCTGGTATCTGC			
CAUD006	ATGGTTCTCTGTAGGCAATC	63.5	183-290	Outbred group
	TTCTGCTTGGGCTCTTGGA			Haplotype
CAUD018	TTAGACAAATGAGGAAATAGTA	50.3	100-180	Outbred group
	GTCCAAACTAAATGCAGGC			Haplotype
CAUD010	GGATGTGTTTTTCATTATTGAT	50.3	138-200	Outbred group
	AGAGGCATAAATACTCAGTG			Haplotype
CAUD012	ATTGCCTTTCAGTGGAGTTTC	63.5	182-286	Outbred group
	CGGCTCTAAACACATGAATG			Haplotype
CAUD014	CACAACTGACGGCACAAAGT	58.1	136-200	Outbred group
	CTGAGTTTTTCCCGCCTCTA			Haplotype
CAUD034	TACTGCATATCACTAGAGGA	55.5	160-296	Outbred group
	TAGGCATACTCGGGTTTAG			Haplotype
CAUD035	GTGCCTAACCCTGATGGATG	63.5	174-282	Outbred group
	CTTATCAGATGGGGCTCGGA			Haplotype
APL579	ATTAGAGCAGGAGTTAGGAGAC	55.0	116-227	Outbred group
	GCAAGAAGTGGCTTTTTTC			Haplotype
AY258	ATGTCTGAGTCCTCGGAGC	58.1	89-162	Outbred group
	ACAATAGATTCCAGATGCTGAA			Haplotype
CMO212	CTCCACTAGAACACAGACATT	58.0	186-272	Outbred group
	CATCTTTGGCATTTTGAAG			Haplotype
CAUD028	TACACCCAAGTTTATTCTGAG	55.5	152-226	Outbred group
	ACTCTCCAGGGCACTAGG			Haplotype
CAUD026	ACGTCACATCACCCCACAG	60.8	134-196	Outbred group
	CTTTGCCTCTGGTGAGGTTC			Haplotype
APH18	TTCTGGCCTGATAGGTATGAG	58.0	178-325	Haplotype
	GAATTGGGTGGTTCATACTGT			
CAUD002	CTTCGGTGCCTGTCTTAGC	60.8	200-231	Haplotype
	AGCTGCCTGGAGAAGGTCT			
CAUD005	CTGGGTTTGGTGGAGCATAA	60.8	184-290	Haplotype
	TACTGGCTGCTTCATTGCTG			

**Table 3:** Number of alleles, optimal amplification conditions, and fragment length of 14 alleles for the outbred colony laboratory geese.

Loci	Primer sequence(5′-3′)	Temperature (°C)	Allele range
G-Ans17	ACAAATAACTGGTTCTAAGCAC	51.0	111–123
	AGAGGACTTCTATTCATAAATA		
G-TTUCG1	CCCTGCTGGTATACCTGA	53.0	113-115
	GTGTCTACACAACAGC		
G-APH13	CAACGAGTGACAATGATAAAA	53.0	163-165
	CAATGATCTCACTCCCAATAG		
G-Ans02	TTCTGTGCAGGGGCGAGTT	58.0	202–230
	AGGGAACCGATCACGACATG		
G-Ans07	GACTGAGGAACTACAATTGACT	62.0	236–246
	ACAAAGACTACTACTGCCAAG		
G-Ans18	GTGTTCTCTGTTTATGATATTAC	56.0	229–237
	AACAGAATTTGCTTGAAACTGC		
G-Ans25	CACTTATTAATGGCACTTGAAA	62.0	261–277
	GTTCTCTTGTCACAACTGGA		
G-Hhi*μ*1b	ATCAAAGGCACAATGTGAAAT	60.0	212–216
	AGTAAGGGGGCTTCCACC		
G-CKW47	AACTTCTGCACCTAAAAACTGTCA	56.0	213-215
	TGCTGAGGTAACAGGAATTAAAA		
G-Bca*μ*5	AGTGTTTCTTTCATCTCCACAAGC	56.0	197-201
	AGACCACAATCGGACCACATATTC		
G-Bca*μ*7	TAGTTTCTATTTGCACCCAATGGAG	60.0	171-175
	CGGTCCTGTCCTTGTGCTGTAA		
G-Bca*μ*8	CCCAAGACTCACAAAACCAGAAAT	58.0	155-159
	ATGAAAGAAGAGTTAAACGTGTGCAA		
G-CAUD006	ATGGTTCTCTGTAGGCAATC	56.0	170-210
	TTCTGCTTGGGCTCTTGGA		
G-APH20	ACCAGCCTAGCAAGCACTGT	53.0	140-150
	GAGGCTTTAGGAGAGATTGAAAAA		

**Table 4:** Number of alleles, optimal amplification conditions, and fragment length of 16 alleles for the outbred colony laboratory pigeons.

Loci	Primer sequence(5′-3′)	Temperature (°C)	Allele range
UU-Cli02	TGGGCAAGGTACACTTTTAGGT	61.0	158-170
	CTTTATGCTCCCCCTTGAGAT		
UU-Cli06	TTTGAAAAACATGGATTGTGC	56.0	140-145
	AATTTGCAGAGGGTGAGTGG		
PG5	GTTCTTGGTGTTGCATGGATGC	59.0	262-266
	AGTTACGAAATGATTGCCAGAAG		
C26L9(1265223)	CAAAGCTGCTGACGTGAATCAA	59.0	467-472
	AGAGACGCTCCATGCAAAAG		
UU-Cli14	CAGAACGTTTTGTTCTGTTTGG	58.0	265-292
	TCTTGCTGCAGTCTTCATCC		
C12L1(532572)	GTTGTTTGGCTGAGTGGACG	62.0	126-136
	TCAACCAGGGGAATTGGCAG		
C12L4(906353)	GCTGCTGTCTTCTTCATTGGG	60.0	210-250
	TTAAAACCTCCCGTCTCCCTG		
Cli*μ*D11	CCAATCCCAAAGAGGATTAT	58.0	78-98
	ACTGTCCTATGGCTGAAGTG		
C26L10(1404758)	GCTGTCAGGTATCAGCCACAA	59.0	211-226
	TCAGACCCACGAAAGCTGTAA		
C26L4(568923)	CAACCCCATGTGGGTGAGAC	63.0	357-432
	CACCACCACGTGGGACATC		
PG4	CCCATCTCCTGCCTGATGC	64.0	136-170
	CACAGCAGGATGCTGCCTGC		
UU-Cli12	CGCCAGACTGTATTGTGAGC	61.0	231-265
	AGCATGGCTGTTCTTTGAGG		
Cli*μ*T47	ATGTGTGTTTGTGCATGAAG	56.0	183-214
	ATGAAAGCCTGTTAGTGGAA		
Cli*μ*D32	GAGCCATTTCAGTGAGTGACA	60.0	136-158
	GTTTGCAGGAGCGTGTAGAGAAGT		
UU-Cli07	GCTGCCTGTTACTACCTGAGC	61.0	277-310
	CTGGCCATGAAATGAACTCC		
C26L1(20390)	AGGAGCCTACACTGGGTTTTC	60.0	250-268
	TGTAGCTCTGCAATCAGCCT		

**Table 5:** Number of alleles, effective alleles, effective heterozygosity, PIC, and Shannon's index of the outbred colony chicken samples.

Loci	Observed number of alleles	Effective number of alleles	Shannon's information index	Effective heterozygosity	PIC
MCW0029	4	2.931	1.209	0.579	0.603
GGNCAMZO	2	1.069	0.146	0.060	0.062
ADL0293	5	3.200	1.311	0.573	0.634
ADL0317	7	5.236	1.768	0.554	0.783
GGAVIR	3	1.916	0.796	0.456	0.408
ADL0201	5	2.103	1.013	0.429	0.482
GCT0016	5	3.042	1.274	0.337	0.618
ADL0304	6	4.641	1.627	0.666	0.751
MCW0402	8	6.042	1.881	0.702	0.813
MCW0063	7	4.319	1.626	0.568	0.736
ADL185	5	3.204	1.359	0.614	0.647
GGMYC	2	1.800	0.637	0.427	0.346
LEI0094	6	3.674	1.468	0.562	0.683
LEI0074	4	3.707	1.348	0.597	0.681
ADL328	3	2.785	1.058	0.526	0.565
GGVITC	1	1.000	0.000	0.000	1.000
GGANTECL	3	2.897	1.080	0.600	0.580
LEI094	6	4.444	1.579	0.690	0.738
MCW0330	4	3.232	1.269	0.577	0.637
LEI0141	4	3.162	1.229	0.341	0.623
ADL0292	3	2.793	1.061	0.475	0.568
GGVITIIG	2	1.965	0.684	0.460	0.371
MCW0087	8	5.930	1.898	0.544	0.810
MCW0347	3	1.948	0.815	0.447	0.419
ADL176	9	4.846	1.858	0.522	0.773
ADL166	5	3.729	1.380	0.574	0.682
MCW0014	5	4.342	1.543	0.592	0.735
GGCYMA	3	1.603	0.632	0.317	0.322
Mean	4.571	3.270	1.198	0.492	0.610

**Table 6:** Number of alleles, effective alleles, effective heterozygosity, and Shannon's index of the haplotype chicken samples.

Loci	Observed number of alleles	Effective number of alleles	Shannon's information index	Effective heterozygosity
GGNCAMZO	1	1.000	0.000	0.000
GGAVIR	2	1.923	0.673	0.480
MCW0402	1	1.000	0.000	0.000
MCW0063	1	1.000	0.000	0.000
ADL185	3	2.174	0.898	0.540
GGMYC	1	1.000	0.000	0.000
LEI0094	3	2.778	1.055	0.640
GGVITC	1	1.000	0.000	0.000
ADL0292	2	1.471	0.500	0.320
GGVITIIG	2	2.000	0.693	0.500
ADL166	1	1.000	0.000	0.000
MCW0014	1	1.000	0.000	0.000
GGCYMA	1	1.000	0.000	0.000
STMSGGHU2-1A	2	1.724	0.611	0.420
Mean	1.571	1.434	0.316	0.207

**Table 7:** Number of alleles, effective alleles, effective heterozygosity, PIC, and Shannon's index of outbred colony duck samples.

Loci	Observed number of alleles	Effective number of alleles	Shannon's information index	Effective heterozygosity	PIC
CMO211	8	4.628	1.698	0.764	0.752
CAUD011	9	5.024	1.835	0.799	0.775
CAUD027	9	3.698	1.588	0.654	0.696
APH09	8	4.840	1.728	0.756	0.763
AY314	12	7.285	2.165	0.806	0.848
AY258	9	3.503	1.586	0.700	0.684
CAUD018	4	2.941	1.194	0.640	0.596
CAUD031	8	4.459	1.711	0.730	0.746
CAUD026	7	4.674	1.697	0.750	0.757
CAUD023	7	2.725	1.315	0.584	0.591
CMO212	8	4.154	1.642	0.739	0.724
CAUD006	4	3.333	1.280	0.440	0.645
CAUD004	7	5.556	1.834	0.720	0.798
CAUD001	6	5.000	1.696	0.600	0.772
CAUD034	10	3.943	1.742	0.730	0.723
CAUD007	8	3.894	1.639	0.714	0.713
APL579	7	3.068	1.412	0.635	0.636
CAUD010	6	4.655	1.630	0.768	0.753
CAUD028	5	3.549	1.378	0.541	0.668
CAUD012	7	3.122	1.354	0.652	0.630
CAUD035	10	5.768	1.922	0.759	0.804
CAUD014	9	3.600	1.448	0.696	0.672
CAUD032	14	6.159	2.120	0.797	0.821
APH11	2	1.923	0.673	0.479	0.365
APL2	4	2.556	1.067	0.609	0.529
Mean	7.520	4.162	1.574	0.683	0.698

**Table 8:** Number of alleles, effective alleles, effective heterozygosity, and Shannon's index of haplotype duck samples.

Loci	Observed number of alleles	Effective number of alleles	Shannon's information index	Effective heterozygosity
CAUD002	3	2.020	0.857	0.360
CAUD006	4	2.740	1.142	0.540
CAUD018	3	1.802	0.746	0.400
CAUD005	5	3.945	1.490	0.551
APL579	5	2.632	1.205	0.500
APH18	7	4.301	1.655	0.640
CAUD010	3	2.597	1.010	0.420
CAUD028	2	1.980	0.688	0.360
CAUD012	3	2.597	1.010	0.420
CAUD035	4	3.756	1.353	0.605
CAUD014	4	3.509	1.306	0.580
CAUD026	4	2.740	1.142	0.520
CMO212	5	3.774	1.458	0.640
AY258	4	2.353	1.063	0.500
CAUD034	6	2.198	1.164	0.460
Mean	4.133	2.863	1.153	0.500

**Table 9:** Number of alleles, effective alleles, effective heterozygosity, PIC, and Shannon's index of outbred colony goose samples.

Loci	Observed number of alleles	Effective number of alleles	Shannon's information index	Effective heterozygosity	PIC
G-Ans17	4	1.843	0.775	0.441	0.388
G-TTUCG1	3	2.255	0.943	0.381	0.494
G-APH13	4	1.605	0.752	0.315	0.352
G-Ans02	8	5.389	1.837	0.749	0.790
G-Ans07	4	3.073	1.220	0.634	0.613
G-Ans18	3	2.208	0.922	0.309	0.481
G-Ans25	4	3.333	1.282	0.629	0.647
G-Hhi*μ*1b	4	2.965	1.147	0.471	0.594
G-CKW47	4	3.143	1.238	0.573	0.623
G-Bca*μ*5	3	2.728	1.051	0.469	0.562
G-Bca*μ*7	6	2.731	1.158	0.455	0.562
G-Bca*μ*8	7	2.845	1.290	0.635	0.599
G-CAUD006	4	3.704	1.344	0.602	0.680
G-APH20	8	4.713	1.772	0.734	0.761
Mean	4.714	3.038	1.195	0.528	0.582

**Table 10:** Number of alleles, effective alleles, effective heterozygosity, PIC, and Shannon's index of outbred colony pigeon samples.

Loci	Observed number of alleles	Effective number of alleles	Shannon's information index	Effective heterozygosity	PIC
UU-Cli02	5	3.613	1.374	0.694	0.672
UU-Cli06	4	2.921	1.163	0.383	0.593
PG5	2	1.681	0.595	0.397	0.323
C26L9(1265223)	4	2.576	1.076	0.602	0.533
UU-Cli14	10	5.144	1.923	0.787	0.784
C12L1(532572)	4	2.810	1.118	0.487	0.575
C12L4(906353)	11	6.375	2.052	0.766	0.825
Cli*μ*D11	7	4.541	1.682	0.734	0.750
C26L10(1404758)	11	9.118	2.281	0.860	0.880
C26L4(568923)	13	5.854	2.062	0.807	0.812
PG4	10	6.847	2.017	0.767	0.836
UU-Cli12	8	2.825	1.364	0.623	0.599
Cli*μ*T47	7	3.492	1.413	0.658	0.666
Cli*μ*D32	9	6.695	1.991	0.807	0.833
UU-Cli07	5	1.352	0.592	0.252	0.251
C26L1(20390)	16	7.014	2.244	0.759	0.844
Mean	7.875	4.554	1.559	0.649	0.674

**Table 11:** Comparison of mean observed allele number, mean effective allele number, mean Shannon's index, and mean effective heterozygosity among the outbred colonies of chickens.

Colonies	Mean observed number of alleles	Mean effective number of alleles	Mean Shannon's information index	Mean effective heterozygosity
BWEL	2.857	2.024	0.730	0.424
BM	2.857	2.132	0.802	0.485
Beijing oil chicken	4.464	2.821	1.088	0.569

**Table 12:** Comparison of mean observed allele number, mean effective allele number, mean Shannon's index, and mean effective heterozygosity among the haplotype chickens.

Colonies	Mean observed number of alleles	Mean effective number of alleles	Mean Shannon's information index	Mean effective heterozygosity
G1	1.571	1.434	0.316	0.207
G2	1.643	1.409	0.335	0.224
G7	2.000	1.626	0.548	0.364

**Table 13:** Comparison of mean observed allele number, mean effective allele number, mean Shannon's index, and mean effective heterozygosity among the outbred colonies of ducks.

Colonies	Mean observed number of alleles	Mean effective number of alleles	Mean Shannon's information index	Mean effective heterozygosity
1	6.320	3.518	1.410	0.685
JD	5.280	3.466	1.335	0.680

**Table 14:** Comparison of mean observed allele number, mean effective allele number, mean Shannon's index, and mean effective heterozygosity among the haplotype ducks.

Colonies	Mean observed number of alleles	Mean effective number of alleles	Mean Shannon's information index	Mean effective heterozygosity
A	2.400	2.022	0.760	0.489
B	2.333	2.029	0.745	0.484
C	2.400	1.912	0.726	0.459
D	2.333	1.944	0.701	0.442

**Table 15:** Comparison of mean observed allele number, mean effective allele number, mean Shannon's index, and mean effective heterozygosity among the outbred colonies of geese.

Colonies	Mean observed number of alleles	Mean effective number of alleles	Mean Shannon's information index	Mean effective heterozygosity
Guangdong Wuzong	4.000	2.769	1.112	0.618
Yangzhou	3.714	2.155	0.802	0.439

**Table 16:** Comparison of mean observed allele number, mean effective allele number, mean Shannon's index, and mean effective heterozygosity among the outbred colonies of pigeons.

Colonies	Mean observed number of alleles	Mean effective number of alleles	Mean Shannon's information index	Mean effective heterozygosity
Silver king	6.125	3.260	1.307	0.647
White king	7.375	4.247	1.435	0.651

## Data Availability

All data, models, and code generated or used during the study appear in the submitted article.

## Supplementary Materials

Supplementary Materials Supplementary Table 1: number of alleles, effective alleles, effective heterozygosity, and Shannon's index of the G1 haplotype chicken population. Supplementary Table 2: number of alleles, effective alleles, effective heterozygosity, and Shannon's index of the G2 haplotype chicken population. Supplementary Table 3: number of alleles, effective alleles, effective heterozygosity, and Shannon's index of the G7 haplotype chicken population. Supplementary Table 4: number of alleles, effective alleles, effective heterozygosity, and Shannon's index of the A haplotype duck population. Supplementary Table 5: number of alleles, effective alleles, effective heterozygosity, and Shannon's index of the B haplotype duck population. Supplementary Table 6: number of alleles, effective alleles, effective heterozygosity, and Shannon's index of the C haplotype duck population. Supplementary Table 7: number of alleles, effective alleles, effective heterozygosity, and Shannon's index of the D haplotype duck population.
