# Correction to “Development of Microsatellite Marker System to Determine the Genetic Diversity of Experimental Chicken, Duck, Goose, and Pigeon Populations”

**DOI:** 10.1155/bmri/9835281

**Published:** 2026-09-09

X. Zhang, Y. He, W. Zhang, Y. Wang, X. Liu, A. Cui, Y. Gong, J. Lu, X. Liu, X. Huo, J. Lv, M. Guo, X. Du, L. Han, H. Chen, J. Chen, C. Li, Z. Chen, “Development of Microsatellite Marker System to Determine the Genetic Diversity of Experimental Chicken, Duck, Goose, and Pigeon Populations,” *BioMed Research International*, 2021, 8851888, https://doi.org/10.1155/2021/8851888

In the article, pigeon‐related data from the author′s previous work [1] is presented and used, without attribution or citation. The authors wish to clarify to the readers that this data was previously published.

We apologize for this error.
