# Supplementary material for: Development of Microsatellite Marker System to Determine the Genetic Diversity of Experimental Chicken, Duck, Goose, and Pigeon Populations
Source: Biomed Res Int. 2021 Jan 14;2021:8851888. doi: 10.1155/2021/8851888 (PMC7822670; doi:10.1155/2021/8851888)
Supplement: Supplementary Materials — Supplementary Table 1: number of alleles, effective alleles, effective heterozygosity, and Shannon's index of the G1 haplotype chicken population. Supplementary Table 2: number of alleles, effective alleles, effective heterozygosity, and Shannon's index of the G2 haplotype chicken population. Supplementary Table 3: number of alleles, effective alleles, effective heterozygosity, and Shannon's index of the G7 haplotype chicken population. Supplementary Table 4: number of alleles, effective alleles, effective heterozygosity, and Shannon's index of the A haplotype duck population. Supplementary Table 5: number of alleles, effective alleles, effective heterozygosity, and Shannon's index of the B haplotype duck population. Supplementary Table 6: number of alleles, effective alleles, effective heterozygosity, and Shannon's index of the C haplotype duck population. Supplementary Table 7: number of alleles, effective alleles, effective heterozygosity, and Shannon's index of the D haplotype duck population. [file 8851888.f1.docx]

| Supplementary Table1 Number of alleles，effective alleles，effective heterozygosity and Shannon’s index of the G1 haplotype chicken population | | | | |  |
| --- | --- | --- | --- | --- | --- |
|  |  |  |  |  |  |
| Loci | Observed number of alleles | Effective number of alleles | Shannon’s Information index | Effective heterozygosity |  |
| GGNCAMZO | 1 | 1.000 | 0.000 | 0.000 |  |
| GGAVIＲ | 2 | 1.923 | 0.673 | 0.480 |  |
| MCW0402 | 1 | 1.000 | 0.000 | 0.000 |  |
| MCW0063 | 1 | 1.000 | 0.000 | 0.000 |  |
| ADL185 | 3 | 2.174 | 0.898 | 0.540 |  |
| GGMYC | 1 | 1.000 | 0.000 | 0.000 |  |
| LEI0094 | 3 | 2.778 | 1.055 | 0.640 |  |
| GGVITC | 1 | 1.000 | 0.000 | 0.000 |  |
| ADL0292 | 2 | 1.471 | 0.500 | 0.320 |  |
| GGVITIIG | 2 | 2.000 | 0.693 | 0.500 |  |
| ADL166 | 1 | 1.000 | 0.000 | 0.000 |  |
| MCW0014 | 1 | 1.000 | 0.000 | 0.000 |  |
| GGCYMA | 1 | 1.000 | 0.000 | 0.000 |  |
| STMSGGHU2-1A | 2 | 1.724 | 0.611 | 0.420 |  |
| Mean | 1.571 | 1.434 | 0.316 | 0.207 |  |

| Supplementary Table2 Number of alleles，effective alleles，effective heterozygosity and Shannon’s index of the G2 haplotype chicken population | | | | |  |
| --- | --- | --- | --- | --- | --- |
|  |  |  |  |  |  |
| Loci | Observed number of alleles | Effective number of alleles | Shannon’s Information index | Effective heterozygosity |  |
| GGNCAMZO | 1 | 1.000 | 0.000 | 0.000 |  |
| GGAVIＲ | 1 | 1.000 | 0.000 | 0.000 |  |
| MCW0402 | 2 | 1.923 | 0.673 | 0.480 |  |
| MCW0063 | 1 | 1.000 | 0.000 | 0.000 |  |
| ADL185 | 3 | 1.515 | 0.639 | 0.340 |  |
| GGMYC | 2 | 1.923 | 0.673 | 0.480 |  |
| LEI0094 | 2 | 2.000 | 0.693 | 0.500 |  |
| GGVITC | 2 | 1.220 | 0.325 | 0.180 |  |
| ADL0292 | 1 | 1.000 | 0.000 | 0.000 |  |
| GGVITIIG | 1 | 1.000 | 0.000 | 0.000 |  |
| ADL166 | 2 | 2.000 | 0.693 | 0.500 |  |
| MCW0014 | 1 | 1.000 | 0.000 | 0.000 |  |
| GGCYMA | 2 | 1.923 | 0.673 | 0.480 |  |
| STMSGGHU2-1A | 2 | 1.220 | 0.325 | 0.180 |  |
| Mean | 1.643 | 1.409 | 0.335 | 0.224 |  |

| Supplementary Table3 Number of alleles，effective alleles，effective heterozygosity and Shannon’s index of the G7 haplotype chicken population | | | | |  |
| --- | --- | --- | --- | --- | --- |
|  |  |  |  |  |  |
| Loci | Observed number of alleles | Effective number of alleles | Shannon’s Information index | Effective heterozygosity |  |
| GGNCAMZO | 1 | 1.000 | 0.000 | 0.000 |  |
| GGAVIＲ | 2 | 1.600 | 0.562 | 0.375 |  |
| MCW0402 | 2 | 1.800 | 0.637 | 0.444 |  |
| MCW0063 | 3 | 2.000 | 0.868 | 0.500 |  |
| ADL185 | 2 | 1.600 | 0.562 | 0.375 |  |
| GGMYC | 2 | 2.000 | 0.693 | 0.500 |  |
| LEI0094 | 2 | 1.600 | 0.562 | 0.375 |  |
| GGVITC | 2 | 1.280 | 0.377 | 0.219 |  |
| ADL0292 | 2 | 1.800 | 0.637 | 0.444 |  |
| GGVITIIG | 2 | 1.600 | 0.562 | 0.375 |  |
| ADL166 | 2 | 1.280 | 0.377 | 0.219 |  |
| MCW0014 | 2 | 1.800 | 0.637 | 0.444 |  |
| GGCYMA | 2 | 1.800 | 0.637 | 0.444 |  |
| STMSGGHU2-1A | 2 | 1.600 | 0.562 | 0.375 |  |
| Mean | 2.000 | 1.626 | 0.548 | 0.364 |  |

| Supplementary Table4 Number of alleles，effective alleles，effective heterozygosity and Shannon’s index of the A haplotype duck population | | | | |  |
| --- | --- | --- | --- | --- | --- |
|  |  |  |  |  |  |
| Loci | Observed number of alleles | Effective number of alleles | Shannon’s Information index | Effective heterozygosity |  |
| CAUD002 | 2 | 1.923 | 0.673 | 0.480 |  |
| CAUD006 | 3 | 2.273 | 0.950 | 0.560 |  |
| CAUD018 | 2 | 1.923 | 0.673 | 0.480 |  |
| CAUD005 | 2 | 2.000 | 0.693 | 0.500 |  |
| APL579 | 2 | 1.471 | 0.500 | 0.320 |  |
| APH18 | 3 | 2.273 | 0.950 | 0.560 |  |
| CAUD010 | 3 | 2.273 | 0.950 | 0.560 |  |
| CAUD028 | 2 | 1.471 | 0.500 | 0.320 |  |
| CAUD012 | 3 | 2.273 | 0.950 | 0.560 |  |
| CAUD035 | 2 | 1.923 | 0.673 | 0.480 |  |
| CAUD014 | 2 | 2.000 | 0.693 | 0.500 |  |
| CAUD026 | 2 | 1.471 | 0.500 | 0.320 |  |
| CMO212 | 3 | 2.273 | 0.950 | 0.560 |  |
| AY258 | 2 | 2.000 | 0.693 | 0.500 |  |
| CAUD034 | 3 | 2.778 | 1.055 | 0.640 |  |
| Mean | 2.400 | 2.022 | 0.760 | 0.489 |  |

| Supplementary Table5 Number of alleles，effective alleles，effective heterozygosit and Shannon’s index of the B haplotype duck population | | | | |  |
| --- | --- | --- | --- | --- | --- |
|  |  |  |  |  |  |
| Loci | Observed number of alleles | Effective number of alleles | Shannon’s Information index | Effective heterozygosity |  |
| CAUD002 | 2 | 1.923 | 0.673 | 0.480 |  |
| CAUD006 | 3 | 2.778 | 1.055 | 0.640 |  |
| CAUD018 | 2 | 1.923 | 0.673 | 0.480 |  |
| CAUD005 | 2 | 1.471 | 0.500 | 0.320 |  |
| APL579 | 2 | 1.600 | 0.562 | 0.375 |  |
| APH18 | 3 | 2.778 | 1.055 | 0.640 |  |
| CAUD010 | 2 | 1.923 | 0.673 | 0.480 |  |
| CAUD028 | 2 | 1.923 | 0.673 | 0.480 |  |
| CAUD012 | 2 | 1.923 | 0.673 | 0.480 |  |
| CAUD035 | 3 | 2.273 | 0.950 | 0.560 |  |
| CAUD014 | 2 | 1.923 | 0.673 | 0.480 |  |
| CAUD026 | 3 | 2.273 | 0.950 | 0.560 |  |
| CMO212 | 3 | 2.778 | 1.055 | 0.640 |  |
| AY258 | 2 | 1.471 | 0.500 | 0.320 |  |
| CAUD034 | 2 | 1.471 | 0.500 | 0.320 |  |
| Mean | 2.333 | 2.029 | 0.745 | 0.484 |  |

| Supplementary Table6 Number of alleles，effective alleles，effective heterozygosity and Shannon’s index of the C haplotype duck population | | | | |  |
| --- | --- | --- | --- | --- | --- |
|  |  |  |  |  |  |
| Loci | Observed number of alleles | Effective number of alleles | Shannon’s Information index | Effective heterozygosity |  |
| CAUD002 | 2 | 1.923 | 0.673 | 0.480 |  |
| CAUD006 | 2 | 1.923 | 0.673 | 0.480 |  |
| CAUD018 | 2 | 1.471 | 0.500 | 0.320 |  |
| CAUD005 | 2 | 1.471 | 0.500 | 0.320 |  |
| APL579 | 3 | 2.273 | 0.950 | 0.560 |  |
| APH18 | 3 | 2.273 | 0.950 | 0.560 |  |
| CAUD010 | 2 | 1.471 | 0.500 | 0.320 |  |
| CAUD028 | 2 | 1.471 | 0.500 | 0.320 |  |
| CAUD012 | 2 | 1.471 | 0.500 | 0.320 |  |
| CAUD035 | 3 | 2.273 | 0.950 | 0.560 |  |
| CAUD014 | 3 | 2.273 | 0.950 | 0.560 |  |
| CAUD026 | 3 | 2.273 | 0.950 | 0.560 |  |
| CMO212 | 3 | 2.273 | 0.950 | 0.560 |  |
| AY258 | 2 | 1.923 | 0.673 | 0.480 |  |
| CAUD034 | 2 | 1.923 | 0.673 | 0.480 |  |
| Mean | 2.400 | 1.912 | 0.726 | 0.459 |  |

| Supplementary Table7 Number of alleles，effective alleles，effective heterozygosity and Shannon’s index of the D haplotype duck population | | | | |  |
| --- | --- | --- | --- | --- | --- |
|  |  |  |  |  |  |
| Loci | Observed number of alleles | Effective number of alleles | Shannon’s Information index | Effective heterozygosity |  |
| CAUD002 | 1 | 1.000 | 0.000 | 0.000 |  |
| CAUD006 | 2 | 1.923 | 0.673 | 0.480 |  |
| CAUD018 | 2 | 1.471 | 0.500 | 0.320 |  |
| CAUD005 | 3 | 2.667 | 1.040 | 0.625 |  |
| APL579 | 3 | 2.273 | 0.950 | 0.560 |  |
| APH18 | 3 | 2.273 | 0.950 | 0.560 |  |
| CAUD010 | 2 | 1.471 | 0.500 | 0.320 |  |
| CAUD028 | 2 | 1.471 | 0.500 | 0.320 |  |
| CAUD012 | 2 | 1.471 | 0.500 | 0.320 |  |
| CAUD035 | 2 | 1.923 | 0.673 | 0.480 |  |
| CAUD014 | 2 | 1.923 | 0.673 | 0.480 |  |
| CAUD026 | 3 | 2.778 | 1.055 | 0.640 |  |
| CMO212 | 3 | 2.778 | 1.055 | 0.640 |  |
| AY258 | 3 | 2.273 | 0.950 | 0.560 |  |
| CAUD034 | 2 | 1.471 | 0.500 | 0.320 |  |
| Mean | 2.333 | 1.944 | 0.701 | 0.442 |  |
